# Optimal ferrofluids for magnetic cooling devices

**DOI:** 10.1038/s41598-021-03514-2

**Published:** 2021-12-17

**Authors:** M. S. Pattanaik, V. B. Varma, S. K. Cheekati, V. Chaudhary, R. V. Ramanujan

**Affiliations:** 1 School of Materials Science and Engineering, Nanyang Technological University, Singapore, 639798 Singapore; 2 Singapore-HUJ Alliance for Research and Enterprise (SHARE), Nanomaterials for Energy and Energy-Water Nexus (NEW), Campus for Research Excellence and Technological Enterprise (CREATE), Singapore, 138602 Singapore

**Keywords:** Nanofluidics, Magnetic properties and materials, Nanoparticles, Fluidics, Fluid dynamics

## Abstract

Superior passive cooling technologies are urgently required to tackle device overheating, consequent performance degradation, and service life reduction. Magnetic cooling, governed by the thermomagnetic convection of a ferrofluid, is a promising emerging passive heat transfer technology to meet these challenges. Hence, we studied the performance metrics, non-dimensional parameters, and thermomagnetic cooling performance of various ferrite and metal-based ferrofluids. The magnetic pressure, friction factor, power transfer, and exergy loss were determined to predict the performance of such cooling devices. We also investigated the significance of the magnetic properties of the nanoparticles used in the ferrofluid on cooling performance. γ-Fe_2_O_3_, Fe_3_O_4_, and CoFe_2_O_4_ nanoparticles exhibited superior cooling performance among ferrite-based ferrofluids. FeCo nanoparticles had the best cooling performance for the case of metallic ferrofluids. The saturation magnetization of the magnetic nanoparticles is found to be a significant parameter to enhance heat transfer and heat load cooling. These results can be used to select the optimum magnetic nanoparticle-based ferrofluid for a specific magnetic cooling device application.

## Introduction

Inefficient use of energy is a major challenge that aggravates the global energy crisis. A significant percentage of the energy supplied to devices and systems is lost to the surroundings as waste heat^1^. According to the energy consumption data from Lawrence Livermore National Laboratory, a significant fraction of overall energy is wasted due to inefficient processes^2,3^.

Removal of waste heat can increase reliability, efficiency, and service life. Several important industrial sectors, e.g., electrical, electronics, mechanical, and transportation, demand better heat transfer solutions^4,5^. One of the technique to improve the heat transfer is by increasing the surface area of heat transfer between the heat load and the cooling device, however it results in a much bulkier cooling device^6^. Hence, there is an urgent need to improve the coolant fluid medium.

Liquid cooling systems are either active or passive^7^. Active cooling systems generally cool heat loads faster than passive cooling systems. However, the major disadvantage of an active cooling mechanism is its requirement of external power, which results in higher cost, vibration, noise, and maintenance^7,8^. On the other hand, passive cooling systems require low or no external energy, are silent, vibration-free, and require low maintenance^7^. The driving force in such passive systems results from the difference in the temperature-dependent properties of fluid or material under the thermal gradient arising from the difference in the waste heat temperature and room temperature^9,10^. Passive cooling systems include heat pipes^11–13^, phase change material-based systems^14–16^, and cooling deploying nanofluids^17^.

Conventional heat pipes are widely used as passive heat transfer systems due to their large effective thermal conductivity^18^ and are governed by the evaporation–condensation cycle principle. However, heat transfer in the conventional heat pipes is limited by pressure fluctuations, flow choking at high vapor velocity, complex geometrical constraints, start-up failure, entrainment limit, viscous limit, gravity dependence, occasional boiling in the wicking structure, and sensitivity of the evaporator to the heat load power and temperature^18,19^.

Hence, new strategies are needed to remove the waste heat effectively. Better management of energy is a crucial factor for sustainable development^20^. Nanofluid cooling is a passive method with high heat transfer values due to the improved thermal conductivity of the coolant^21^. Nanofluids are stable suspensions of metallic or non-metallic nanoparticles in a carrier liquid medium^5^. The high surface-to-volume ratio of nanoparticles gives rise to better stability of the suspension and increases the effective thermal conductivity of the coolant medium. A subclass of nanofluids is known as ferrofluid.

A ferrofluid is a stable colloidal suspension of nanometer-sized magnetic particles in a carrier fluid^22^. These magnetic nanoparticles (MNP) are usually ferrites or alloys of iron, nickel, and cobalt. Ferrofluids possess both fluidic and magnetic behavior, which provides the advantage of controlling ferrofluid motion by an external magnetic field^23,24^. A stable ferrofluid usually implies that the size of the suspended MNP should be less than 15 nm in diameter^25^. Under the influence of a magnetic field, the MNP orient along the magnetic field direction, and the ferrofluid behaves like a liquid magnet. The extent of ferrofluid magnetization depends on the type (metallic, alloy, or ferrite) and concentration of MNP constituents in the ferrofluid, the strength and orientation of the applied magnetic field, and the temperature of the ferrofluid. Ferrofluid motion can be controlled by changing the magnitude and direction of the external magnetic field. Since the ferrofluid can be remotely manipulated in a wireless fashion, it has a multitude of applications in targeted drug delivery^26,27^, hyperthermia^28–30^, droplet microfluidics^31,32^, particle synthesis^33^, energy harvesting^5,34^ and waste heat recovery^35–37^.

Ferrofluid flow, under the combined effect of both temperature and magnetic field gradients, can be controlled by an external magnetic field. This phenomenon is called thermomagnetic convection^23^ and is used to develop passive magnetic heat transfer systems. Moreover, magnetic cooling devices based on the thermomagnetic effect are noise-free, vibration-free, require no or low maintenance, and are self-pumping and self-regulating. The use of ferrofluids as a coolant results in a thermal conductivity enhancement of ~ 300% under an external magnetic field^38,39^.

**Figure 1:**
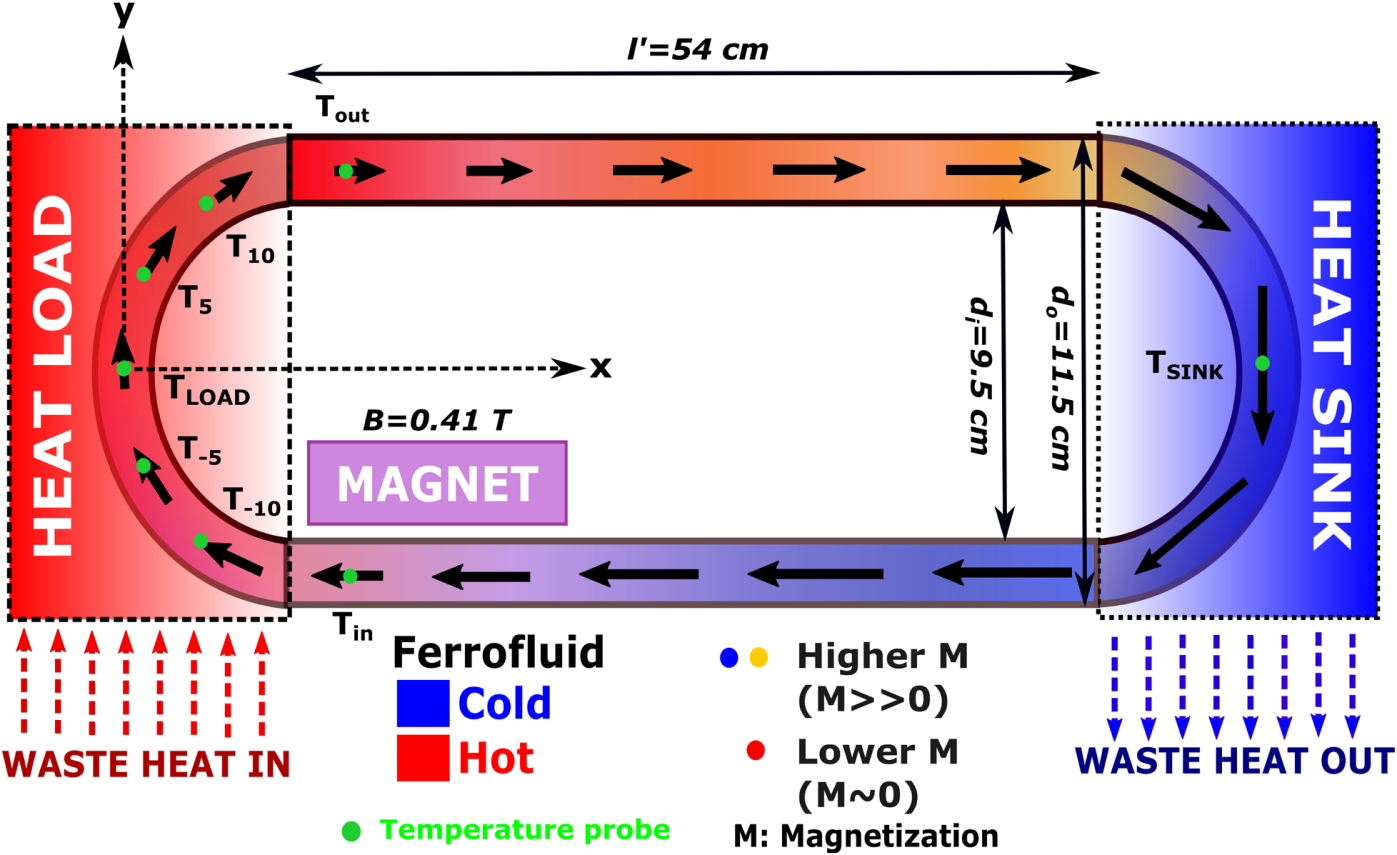
Schematic of a copper-based racetrack magnetic cooling device, governed by the ferrofluid’s thermomagnetic convection. A ferrofluid flows in the closed-loop under the combined effect of thermal gradient and magnetization gradient. An NdFeB permanent magnet with a saturation magnetic flux density of 0.41 T provides the necessary magnetic field. The direction of arrows indicates the flow direction. The length of the arrow and the color of the fluid represents the relative ferrofluid magnetization. Green dots denote the location of T-type thermocouples placed on heat load and heat sink surface. T_i_ with i = -10 to 10 denotes temperature probe positions. |i| is the arc length in cm from the origin. In addition, the dimensions of the magnetic cooling device are provided. Notations: T_LOAD_ (heat load temperature), T_SINK_ (heat sink temperature), T_i_ (temperature of the heat load at position i, i is the distance of the probe from the origin), Tin (inlet temperature), Tout (outlet temperature), l′ (inflow/outflow straight channel length), d_i_/d_o_ (inner/outer diameter of the semi-circular heat load and heat sink arc).

In a magnetic cooling device, the thermal gradient arising from the difference between the heat load and heat sink temperatures results in a magnetization gradient in the ferrofluid column near the heat load region. As a result, the ferrofluid at the heat load possesses low or zero magnetization, whereas the ferrofluid near the heat sink possesses higher magnetization. As a result, the differentially heated ferrofluid starts to flow around the closed loop in the presence of an external magnetic field due to the non-uniform magnetic volume force. A schematic of such a device is presented in Fig. 1.

However, the passive nature of magnetic cooling devices depends on the type of magnetic field used.

A. AC magnetic field such as an electromagnet requires external energy input. Hence, any magnetic cooling device using an AC magnetic field is a passive cooling system with low external energy.
B. On the other hand, using a permanent magnet as a magnetic field source does not require any external energy input. Hence, magnetic cooling devices that use permanent magnets are categorized as truly passive cooling systems, requiring no external energy.

The heat load cooling using a magnetic cooling device depends on various parameters, as stated below.

**Table:** 

**A. Ferrofluid parameters:**	
1. Magnetic Properties	Bulk saturation magnetization, initial magnetic susceptibility, pyro magnetic coefficient, Curie temperature, MNP volume fraction
2. Thermophysical Properties	Viscosity, thermal conductivity, specific heat capacity, type of ferrofluid: binary/ hybrid
**B. Device parameters:**	
3. Dimensions	Device length, channel diameter, device footprint, aspect ratio
4. Properties	Thermal conductivity, specific heat capacity
5. Orientation	Horizontal, inclined, vertical
C. Other parameters:	
6. Magnetic field	Strength, orientation, position, uniform/non-uniform, number of magnets, frequency of AC magnetic field
7. Heat load	Temperature, power, heat flux, no. of heat loads

Magnetic cooling devices of various length scales viz., microscale (mm length-scale, mW power), small-scale (cm length-scale, W power), and large-scale (m length-scale, kW power), were developed and investigated for their thermomagnetic cooling performance. Earlier investigations mainly examined the cooling of microscale devices, e.g., for MEMS and LOC applications^40–43^. Small scale devices were researched to cool power electronics and small mechanical systems. Lian et al.^44^ performed particle image velocimetry (PIV) measurements to determine ferrofluid velocity for several heat loads and flow channel geometries. Li et al.^45^ developed a rectangular cross-section magnetic cooling device to examine the effect of external magnetic field strength and heat load power on the ferrofluid flow and heat transfer characteristics.

Recent studies focused on magnetic cooling devices with meter size flow channel length and larger channel diameter for long-distance and high-power waste heat transfer. Pattanaik et al.^46^ performed kW level cooling of heat load by a multi-torus magnetic cooling device. The device was with a 1.8 m long effective channel, 25 mm channel diameter, and used a commercial oil-based Fe_3_O_4_ ferrofluid as a working fluid. Khairul et al.^17^ investigated magnetic field effects on thermomagnetic convection for laminar, turbulent flow conditions for a water-based magnetite ferrofluid. Yamaguchi et al.^47^ used a temperature-sensitive binary magnetic fluid to develop a 5 m perimeter magnetic cooling system.

Goharkhah et al.^21^ modeled a ferrofluid's forced thermomagnetic heat transfer under the influence of eight AC magnetic field line source dipoles. Fadaei et al.^48^ numerically investigated the effect of magnetic field rotation on cooling performance. Hybrid ferrofluids were also investigated to improve the thermomagnetic cooling performance. Shahsavar et al.^49^ experimentally investigated the heat transfer performance of CNT-loaded ferrofluid in the presence of constant as well as alternating magnetic fields. Sadeghinezhad et al.^50^ experimentally studied the improvement in convective heat transfer using a graphene-loaded ferrofluid under an external magnetic field.

There are a few reports focused on the thermomagnetic cooling performance of ferrites- and metallic-based ferrofluids. Love et al.^40^ designed and investigated a magnetocaloric pump for lab-on-chip applications. They synthesized oil-based magnetite and water and oil-based Mn-Zn ferrite ferrofluids and compared their cooling in a magnetic cooling system. Chaudhary et al. investigated the magnetic cooling performance of a water-based Mn-Zn ferrite ferrofluid using both experiments and simulations. They investigated the effect of heat load temperature, the concentration of magnetic nanoparticles in the ferrofluid, and saturation magnetization of the magnetic nanoparticles on the cooling performance. A similar research work using Mn-Zn ferrite was carried out by Phor et al.^51^. Sharma et al.^37^ synthesized Fe–Cr-Al magnetocaloric ferrofluids and showed the effect of chromium content in Fe–Cr-Al, magnetic nanoparticle volume fraction, and heat load power on magnetic cooling.

These reports investigated the cooling performance as a function of device properties, heat load parameters, magnetic field strength, position, and orientation. Most of the literature described the use of ferrite-based commercial ferrofluids. For enhanced heat load cooling, Aursand et al.^52^ numerically investigated cooling based on their optimization results for solenoid geometry, power consumption in the solenoid, thermomagnetic pumping, and MNP size.

Despite its apparent critical importance in cooling, there is surprisingly no formal ranking of the cooling performance of various ferrite- or metallic- MNP ferrofluids.

Hence, the scope of the present study is to investigate the cooling performance of the magnetic cooling device for a range of values of the magnetic and thermophysical properties of the ferrofluid. For the first time, we modeled the performance metrics, non-dimensional parameters, and heat load cooling performance to rank ferrite and metallic ferrofluids by their cooling performance. The magnetic pressure term was derived and found to be a function of saturation magnetization, magnetic nanoparticle volume fraction, temperature-dependent initial magnetic susceptibility, and magnetic field strength. The friction factor, power transferred from the heat load to the heat sink, and the exergy loss were derived as a function of magnetic, thermal, and thermophysical parameters. Numerical simulations were performed to examine the effect of magnetic parameters of the nanoparticles viz., bulk saturation magnetization, Curie temperature, pyromagnetic coefficient, and initial magnetic susceptibility on the cooling performance. Several ferrite and metallic ferrofluids were included in this study.

It was found that ferrite-based ferrofluids of γ-Fe_2_O_3_, Fe_3_O_4_, and CoFe_2_O_4_ exhibited superior cooling performance. For the case of metallic ferrofluids, FeCo based ferrofluid exhibited the best cooling performance, followed by Fe and FeNi ferrofluids. The bulk saturation magnetization of the MNP was found to be a significant parameter that enhanced the heat transfer and heat load cooling. Higher thermal conductivity and lower viscosity give rise to higher cooling performance. The Nusselt number, magnetic Rayleigh number, and Peclet number increased significantly for higher bulk saturation magnetization. Our ranking of various ferrofluids can be used to select the optimum ferrofluid for enhanced heat load cooling for a specific application.

## Analytical and modeling methods

 “Numerical model” and “Governing equations for numerical model” sections describe the basics of the numerical model to investigate the thermomagnetic convection of various ferrofluids and the governing equations of the same. “Analytical methods for the derivation of the figure of merit” section presents a simple analytical derivation of the figure of merit using 1D governing equations for the thermomagnetic flow around a closed loop. “Non-dimensional parameters and scaling” section briefs the non-dimensional parameters required to understand heat transfer in magnetic cooling devices. “Boundary condition of the numerical model” and “Numerical verification and validation” sections list all the boundary conditions and validation of the numerical model respectively, which are required to investigate the magnetic cooling by ferrite- and metallic-based ferrofluids. “Parameters considered” section includes all the parameters considered to simulate the thermomagnetic convection effect in this research work.

### Numerical model

COMSOL Multiphysics-based 2D numerical simulations investigated the magnetic cooling performance of various ferrite- and metallic-based ferrofluids for a racetrack device. The ferrofluids were treated as a single-phase continuum as per Rosensweig's continuum approach^25^. Thermal, fluidic, and magnetic properties of the ferrofluids were provided as inputs to the numerical model. Three physics modules were employed, viz., magnetic field, heat transfer, and single-phase flow in the laminar regime. The magnetic volume force was added as an additional volume force term in the momentum conservation equation due to the thermomagnetic convection. Both magnetic volume force and the magnetic field vary non-linearly with distance (r) from the magnet^53^. To avoid any error that might arise from the discretization of magnetic volume force, we defined the corresponding components at the wall of the tube. Dense meshing was used along the boundary to avoid any errors due to the thin boundary layer. The magnetic field distribution of a suitable NdFeB magnet was simulated and compared to the experimentally obtained magnetic field strength values. The boundary conditions were employed as per the experimental design. The 2D numerical model was validated to obtain simulation accuracy.

### Governing equations for numerical model

The following governing equations are used to model the thermomagnetic convection effect. The magnetic field distribution around the NdFeB permanent magnet was given by Maxwell's equations^54–56^:

1 $$\nabla \times {\varvec{H}} = 0$$

2 $${\varvec{H}} = - \nabla V_{m}$$

3 $$\nabla \cdot \,{\varvec{B}} = 0$$

4 $${\varvec{B}} = \mu_{0} \left( {{\varvec{H}} + {\varvec{M}}} \right) = \mu_{0} \left( {1 + \chi_{m} } \right){\varvec{H}}$$

where **H** is the magnetic field, V_m_ is the magnetic scalar potential, **B** is the magnetic flux density. μ_0_ and χ_m_ are the magnetic permeability of free space and magnetic susceptibility of the ferrofluid, respectively.

The non-uniform magnetic susceptibility of the ferrofluid as a function of temperature and the magnetic field is given by the Langevin eqaution^33,55,56^:

5 $$\chi_{m} \left( {H,T} \right) = \frac{{{\mathbb{C}}_{0} M_{s}^{Bulk} }}{H} \times {\mathcal{L}}\left( {\gamma H} \right)$$

where ${\mathbb{C}}_{0}$ is the volume fraction of the magnetic nanoparticles in the ferrofluid, ${\text{M}}_{{\text{s}}}^{{{\text{Bulk}}}}$ is the bulk saturation magnetization of the ferrofluid, and γ is the Langevin parameter.

${\mathcal{L}}$ is the Langevin function, which takes the form:

6 $${\mathcal{L}}\left( {\gamma H} \right) = \coth (\gamma H) - \left( {\frac{1}{{\gamma H}}} \right)$$

The Langevin parameter γ is a function of temperature and is defined as:

7 $$\gamma \left( T \right) = \frac{{3\chi_{i} \left( T \right)}}{{M_{s}^{Bulk} }}$$

where χ_i_ is the initial susceptibility of the magnetic nanoparticles over a range of temperature values below the Curie temperature.

The transient form of the continuity equation, the Navier–Stokes equation, and the heat transfer equation describe mass, momentum, and energy conservation^46^. These transient forms of the mass, momentum, and energy conservation equations are used in the model to simulate the thermomagnetic convection effect.

8 $$\nabla \cdot \,{\varvec{v}} = 0$$

9 $$\rho \left( {\frac{{\partial {\varvec{v}}}}{\partial t} + \left( {{\varvec{v}} \cdot \nabla } \right){\varvec{v}}} \right) = - \nabla p + \nabla \cdot \left( {\eta \left( {\nabla {\varvec{v}} + \left( {\nabla {\varvec{v}}} \right)^{T} } \right)} \right) + {\varvec{F}}_{{\varvec{m}}}$$

10 $$\rho C_{p} \left( {\frac{\partial T}{{\partial t}} + {\varvec{v}} \cdot \nabla T} \right) = \nabla \cdot \left( {\kappa \nabla T} \right)$$

where T, **v**, and p denote temperature, velocity, and pressure of the ferrofluid, respectively. **F**_**m**_ is the non-uniform magnetic volume force acting on the ferrofluid. The ferrofluid’s thermophysical properties are dynamic viscosity (${\upeta }$), density (ρ), specific heat (C_p_), and thermal conductivity (κ).

The magnetic volume force (**F**_**m**_) experienced by the ferrofluid in the presence of an applied magnetic field is given by Pattanaik et al.^46^:

11 $${\varvec{F}}_{{\varvec{m}}} = \frac{{{\mathbb{C}}_{0} \chi_{mnp} }}{{\mu_{0} }}\left( {{\varvec{B}} \cdot \nabla } \right){\varvec{B}} = \frac{{\chi_{m} \left( {H, T} \right)}}{{\mu_{0} }}\left( {{\varvec{B}} \cdot \nabla } \right){\varvec{B}}$$

where, ${\upchi }_{{{\text{mnp}}}}$ represent the volume magnetic susceptibility of the magnetic nanoparticles. ${\upchi }_{{\text{m}}}$ is the magnetic susceptibility of the ferrofluid.

### Analytical methods for the derivation of the figure of merit

**Figure 2:**
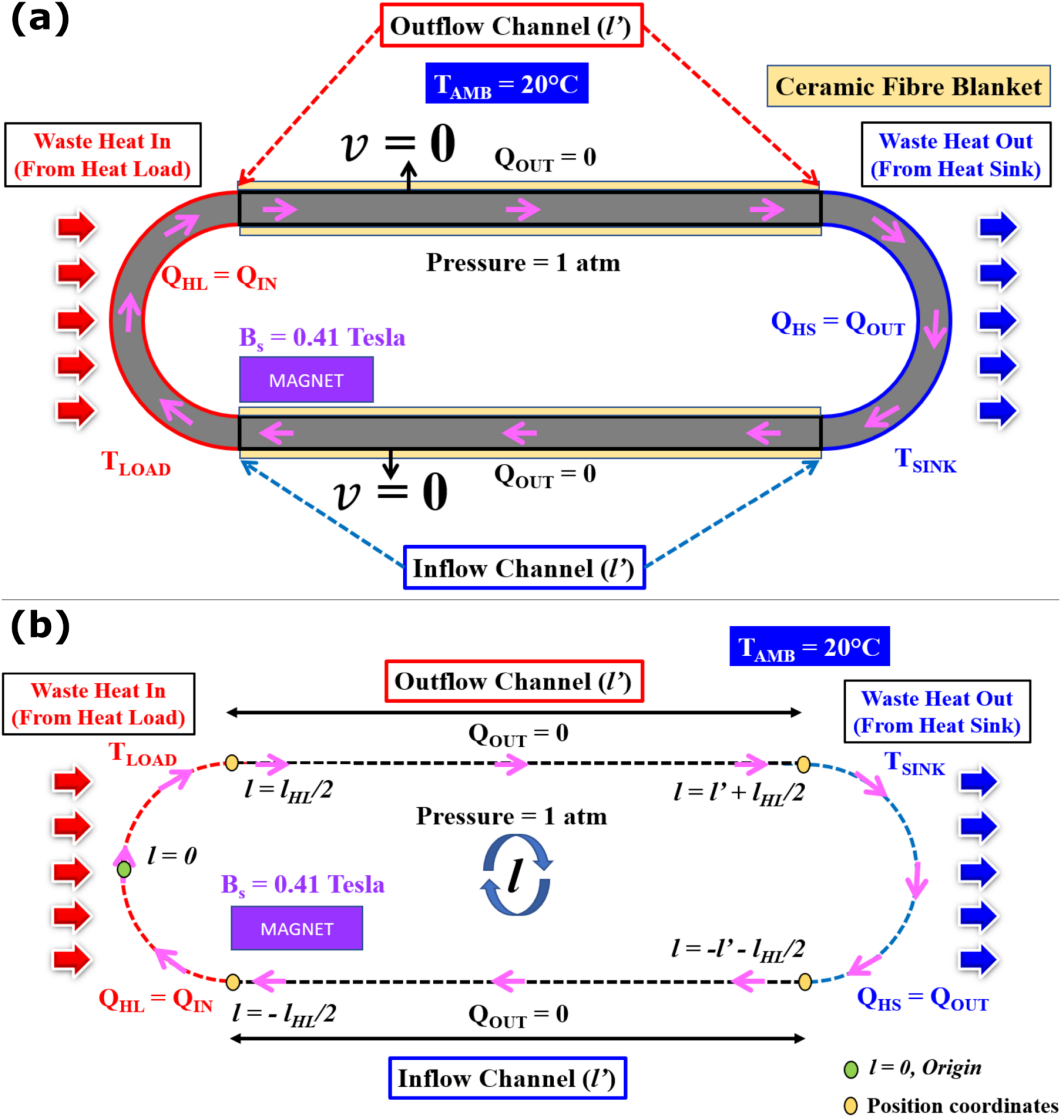
(**a**) Schematic of the boundary conditions used for the simulation of racetrack-shaped magnetic cooling device*. No-slip boundary condition is considered at the flow channel wall. (**b**) Outline of the one-dimensional flow loop approach used for the analytical derivation of temperature, magnetic pressure, and friction factor. The green ellipse denotes the flow loop length (l) origin l = 0. The yellow ellipses denote locations and lengths of the flow loop (l) from the origin required to derive the figure of merit. Pink arrows represent the ferrofluid flow direction along the flow loop. Notations: Q_HL_ (heat load power), Q_IN_ (input power), Q_HS_ (heat sink power), Q_OUT_ (output power), T_LOAD_ (heat load temperature), T_SINK_ (heat sink temperature), T_AMB_ (Ambient temperature), *v* (fluid velocity at the wall), l (flow channel length from the origin), l_HL_ (heat load arc length), l′ (inflow and outflow channel lengths). *Not shown here: heat load insulation by a ceramic fiber blanket wool.

The analytical derivation presented in this section is based on a simple one-dimensional (1-D) approach (Fig. 2). The axis along the middle of the flow loop (l, flow loop length) is taken as the 1-D coordinate to derive the ferrofluid temperature, magnetic pressure (Δp_m_), and the friction factor (f). The schematic of the 1-D flow channel loop that is used to derive the temperature of the ferrofluids at various regions of the flow loop, the magnetic pressure, and the friction factor is shown in Fig. 2a, b. The middle of the heat load section is taken as the origin of l. The l value at various points along the flow loop is labeled in the figure. The magnetic cooling device has 4 different regions: (a) heat load region, (b) outflow channel, (c) heat sink region, and (d) inflow channel, as shown in Fig. 2b. While developing the reduced 1-D governing equations, the cross-sectional average is considered for the thermomagnetic convection. The following assumptions were taken into consideration while deriving for the temperature, magnetic pressure, and friction factor.

- The cross-sectional average temperature of the ferrofluid is similar to the bulk temperature of the ferrofluid along the axial flow loop length (l).
- Axial conduction of heat is negligible compared to convection-based heat transfer.
- The steady-state form of the 1-D conservation equations are considered.
- All the equations considered and the subsequent equations derived are for a given time (t). Hence the time variation is not taken into account.
- Gravitational effects are neglected.

The volume flow rate (q̇) of a ferrofluid within a closed circulation flow loop can be written as

12 $$\dot{q} = Av$$

where A is the cross-sectional area of the flow loop. The steady-state form of the conservation Eqs. (8–10) in terms of flow loop length l can be rewritten from Eq. (12) as,

13 $$\frac{{\partial \dot{q}}}{\partial l} = 0$$

14 $$\frac{\rho }{{A^{2} }}\frac{{\partial \dot{q}^{2} }}{\partial l} = - \frac{\partial p}{{\partial l}} + \frac{\eta }{A}\frac{{\partial^{2} \dot{q}}}{{\partial l^{2} }} + F_{m}$$

15 $$\dot{q}\frac{\partial T}{{\partial l}} = \alpha A\frac{{\partial^{2} T}}{{\partial l^{2} }}$$

Here, α is the thermal diffusivity of the ferrofluid.

The energy conservation equations for the heat load section, heat sink section, and the inflow and outflow channels can be written as,

16 $$\kappa \dot{q}\frac{dT}{{dl}} = \alpha \frac{P}{{l_{HL} }}$$

17 $$\kappa \dot{q}\frac{dT}{{dl}} = - \alpha \frac{{h_{HS} A\left( {T_{f} - T_{w} } \right)}}{{l_{HS} }}$$

18 $$\dot{q}\frac{dT}{{dl}} = 0$$

Here, P is the applied heat load power, and h_HS_ is the heat transfer coefficient at the heat sink. The length of the heat load and the heat sink is denoted as l_HL_ and l_HS_, respectively. T_f_ and T_w_ denote the temperature of the bulk ferrofluid and the flow channel wall, respectively. The right-hand side of the equation is zero because of the negligible temperature difference between the ferrofluid and the flow channel wall and the adiabatic nature of the flow channels. Integrating the above energy equations lead to the following temperature expressions,

19 $$T\left( l \right) = \frac{P}{{l_{HL} \rho C_{p} \dot{q}}}l + T_{in} ,\;for \left( { - \frac{{l_{HL} }}{2} \le l \le \frac{{l_{HL} }}{2}} \right)$$

20 $$T\left( l \right) = T_{out} , \;for \left( {\frac{{l_{HL} }}{2} \le l \le \frac{{l_{HL} }}{2} + l^{\prime}} \right)$$

21 $$T\left( l \right) = T_{HS} + \left( {T_{out} - T_{HS} } \right)exp\left\{ { - \frac{{\pi Nu_{HS} \alpha }}{{\dot{q}}}l} \right\}, \;for \left( {\frac{{l_{HL} }}{2} + l^{\prime} \le l \le - \frac{{l_{HL} }}{2} - l^{\prime}} \right)$$

22 $$T\left( l \right) = T_{in} , for \left( { - \frac{{l_{HL} }}{2} - l^{\prime} \le l \le - \frac{{l_{HL} }}{2}} \right)$$

The above expressions provide the ferrofluid temperature at any point along the flow channel. Equations (19)–(22) are the expressions for evaluating the ferrofluid temperature as a function of the flow loop length (l) at the heat load region, along the outflow channel, heat sink region, and along the inflow channel, respectively. T_in_, T_out_, and T_HS_ are temperatures of ferrofluid at the inlet, the outlet temperature, and the heat sink, respectively. ${\text{l}}^{^{\prime}}$ represents the length of the inflow and outflow channels of the racetrack magnetic cooling device.

The 1D momentum equation (Eq. (14)) can be integrated around the racetrack flow channel to obtain the friction factor as a function of the resultant magnetic pressure due to the thermomagnetic convection effect. Integration of the steady-state momentum equation takes the form,

23 $$\oint {\left( {\frac{\rho }{{A^{2} }}\frac{{\partial \dot{q}^{2} }}{\partial l}} \right)d} l = -\oint {\left( {\frac{\partial p}{{\partial l}}} \right)dl + \oint {\left( {\frac{\eta }{A}\frac{{\partial^{2} \dot{q}}}{{\partial l^{2} }}} \right)dl} } + \oint {\left( {\mu_{0} M\left( {T,H} \right)\frac{\partial H}{{\partial l}}} \right)dl}$$

The integration of the convective acceleration term (1st term in Eq. (23)) is zero owing to the continuity equation (Eq. (13)), under the assumption of the constant density of the ferrofluid across the flow channel. The pressure gradient term around the flow channel vanishes as the pressure difference along a closed loop is zero. Integration of the viscous drag term (3^rd^ term in Eq. (23)) leads to Eq. (24)^57^.

24 $$\int {\frac{\eta }{A}\frac{{\partial^{2} \dot{q}}}{{\partial l^{2} }}} = - \frac{{fl_{d} }}{{d_{t} }}\frac{{\rho \dot{q}^{2} }}{{2A^{2} }}$$

where f is the friction loss associated with the magnetic cooling device. l_d_ and d_t_ are the total perimeter and internal diameter of the flow channel, respectively. Integrating the added magnetic volume force term in the Navier–Stokes equation gives rise to the resultant magnetic pressure (Δp_m_) due to the thermomagnetic convection effect.

25 $$\Delta p_{m} = \oint {F_{m} dl} = \oint {\left( {\mu_{0} M\left( {T,H} \right)\frac{\partial H}{{\partial l}}} \right)dl}$$

Using Eqs. (5), (6) in Eq. (25), we obtain Δp_m_ as a function of T, H, buslk Ms, and ${\mathbb{C}}_{0}$.

26 $$\Delta p_{m} = \mu _{0} \mathbbm{C}_{0} M_{s}^{{bulk}} \left[ {\left\{ {ln\left( {\frac{{\left| {sinh\left( {\gamma H_{{max}} } \right)} \right|}}{{\gamma H_{{max}} }}} \right)^{{\frac{1}{\gamma }}} } \right\}_{{T\left( {l_{{max}} } \right)}} - \left\{ {ln\left( {\frac{{\left| {sinh\left( {\gamma H_{{min}} } \right)} \right|}}{{\gamma H_{{min}} }}} \right)^{{\frac{1}{\gamma }}} } \right\}_{{T\left( {l_{{min}} } \right)}} } \right]$$

H_max_ and H_min_ correspond to the maximum and the minimum field experienced by the ferrofluid in the vicinity of the heat load region. l_max_ and l_min_ are the corresponding positions along the flow channel with respect to the maximum and minimum applied magnetic field, respectively. In the limit of zero magnetic fields,

27 $$\mathop {\lim }\limits_{{H \to 0}} ln\left( {\frac{{\left| {sinh\left( {\gamma H} \right)} \right|}}{{\gamma H}}} \right)^{{\frac{1}{\gamma }}} = 1$$

Hence, the magnetic pressure term is negligible at low applied magnetic fields and vanishes at zero magnetic field. The magnetic pressure term is directly proportional to the ferrofluid's bulk saturation magnetization and magnetic nanoparticle volume fraction. The temperature dependence arises from the Langevin parameter (γ).

Following the substitution of Eqs. (24) and (26) in Eq. (23), we obtain the friction factor (f)^17^,

28 $$f = \frac{{\Delta p_{m} \pi^{2} d_{t}^{5} }}{{8l_{HL} \rho \dot{q}^{2} }}$$

Exergy refers to the maximum useful work a system can perform when it is brought into equilibrium with its surrounding^58,59^. Exergy loss refers to the irreversible lowering of the maximum available work outside the control volume^17^. For thermomagnetic convection based magnetic cooling device, under the assumption of zero energy exchange between the system and the surrounding, the amount of exergy loss at the steady-state of a ferrofluid is given by Khairul et al.^17^,

29 $$P^{^{\prime\prime}} = T_{amb} \left[ {\dot{m}C_{p} ln\left( {\frac{{T_{out} }}{{T_{in} }}} \right)} \right]$$

Here, T_amb_ refers to the ambient temperature.

### Non-dimensional parameters and scaling

To evaluate the thermomagnetic cooling performance and to rank various ferrofluids, we analyzed several non-dimensional parameters viz., Nusselt number (Nu), magnetic Rayleigh number (Ra_m_), Peclet number (Pe), and Stanton number (St). The values of these non-dimensional parameters vs. the bulk saturation magnetization of various ferrite and metallic magnetic nanoparticles can be used to rank the ferrofluids. The detailed information and formulae for these non-dimensional numbers are provided in Section 1 of the Supplementary file.

### Boundary condition of the numerical model

Figure 2a summarizes various boundary conditions used for the 2D numerical simulation. The ferrofluid was assumed to be Newtonian, single-phase, and incompressible fluid^55,56^. At the flow channel walls, a No-slip boundary condition was applied. The heat sink and the ambient surface temperature were set to 20 °C. Heat flux values were specified to simulate heating of the heat load with thermal insulation applied to the other sections of the flow loop. The initial ferrofluid conditions were set to 1 atm pressure and 20 °C room temperature. For magnetic field and fluid flow simulations, a zero magnetic scalar potential condition and a pressure point constraint were employed, respectively.

### Numerical verification and validation

The developed 2D simulation model was numerically verified by performing a mesh independency test to ensure better simulation accuracy using 2D triangular elements-based meshing^55,56^. Dimension-less heat load cooling (ΔT/ΔT_max_) vs. number of mesh elements was plotted at a heat flux value of 1.6 kW/m^2^ (Fig. 1a, Section 2, Supplementary file) for numerical validation. The final model used 249,554 triangular mesh elements and an average mesh quality of 89.67%. Triangular mesh elements divided the entire racetrack geometry of the magnetic cooling device was divided into discrete regions.

The model was also numerically validated by magnetic field simulations (Fig. 1b,c, Section 2, Supplementary file) compared with experiments. The experiments and simulations of the NdFeB magnet’s surface magnetic field agree with an 6.2% absolute error.

### Parameters considered

The effect of magnetic parameters of the ferrofluid, viz., bulk saturation magnetization (${\text{M}}_{{\text{s}}}^{{{\text{Bulk}}}}$), initial magnetic susceptibility (χ_i_), pyromagnetic coefficient (dχ/dT), and Curie temperature (Tc) on the heat load cooling (ΔT) were evaluated. The heat flux (Q) was also varied to study its effect on heat load cooling. The ${\text{M}}_{{\text{s}}}^{{{\text{Bulk}}}}$ and χ_i_ curves for various ferrite and metallic-based ferrofluids were considered for ranking the ferrofluids. Various performance metrics and non-dimensional numbers were studied as a function of ${\text{M}}_{{\text{s}}}^{{{\text{Bulk}}}}$ of the magnetic nanoparticles.

The requirement of a ferrofluid for effective magnetic cooling are as follows.

- Use of soft magnetic nanomaterials with high magnetic permeability and extremely low coercivity for faster response to the external magnetic field
- High pyromagnetic coefficient near the heat load temperature for superior heat load cooling
- High saturation magnetization
- Low magneto-crystalline anisotropy of magnetic nanoparticles^53^
- Low viscosity of the ferrofluid

The first 4 refers to the properties of the magnetic nanoparticles. We have chosen a range of ferrite- and metallic-based nanoparticles based on the criteria. Table 1 summarizes the parameters, their units, notation, and experimental sets considered in this work.

**Table 1:** Parameters, their notation, range, and the measurement sets.

Parameters	Unit	Notation	Performed sets
Bulk saturation magnetization	kA/m	${\text{M}}_{{\text{s}}}^{{{\text{bulk}}}}$	50–2002
Initial magnetic susceptibility	–	χ_i_ (T = 0 °C)	1.75–14
Ratio of pyromagnetic coefficient	–	(dχ″/dT)/(dχ′/dT)	64 to 156
Curie temperature	°C	Tc	75–200
Heat flux	kW/m^2^	Q	0.6–2.6
Initial heat load temperature	°C	T_0_	60–200

## Results and discussions

### Experimental and simulated heat load cooling for magnetite ferrofluid

Before performing a numerical investigation for various ferrofluids, the simulation results were validated against the experimental cooling results obtained from a copper racetrack-shaped magnetic cooling device (Fig. 1) with a perimeter of 130 cm and an internal tube diameter of 0.81 cm. T-type thermocouples were mounted on the surface of the heat load at various locations, as shown in Fig. 1. Data acquisition was performed by a lab-view setup and a data logger (Pico technology, model: TC-08, channels: 8).

**Figure 3:**
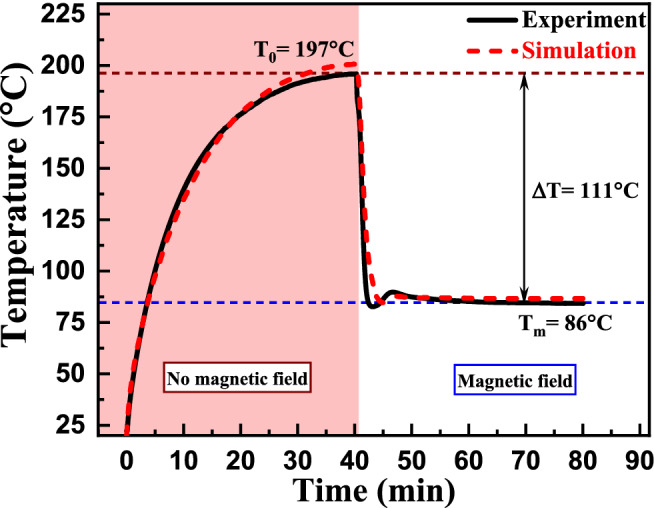
Experimental heat load temperature vs. time curve with and without the application of an external magnetic field. Initial saturated heat load temperature without magnetic field was (T_LOAD_ = T_0_ for B = 0 T) 197 °C. T_m_ is the heat load temperature achieved after an applied magnetic field of 0.41 T (after magnetic cooling).

The experimentally obtained heat load temperature profile was compared with the simulated profile for a heat flux value of 3.49 kW/m^2^ and an initial heat load temperature of 197 °C for a copper-based racetrack magnetic cooling device containing commercial Fe_3_O_4_ ferrofluid. The experimental and simulated temperature profiles are in good agreement (Fig. 3). This simulation was employed to study the effect of ferrofluidic thermophysical and magnetic properties on cooling performance.

### Parametric investigation of heat load cooling

A preliminary study to assess the significance of various magnetic parameters of the ferrofluid viz., initial magnetic susceptibility (χ_i_), pyromagnetic coefficient (dχ/dT), bulk saturation magnetization (${\text{M}}_{{\text{s}}}^{{{\text{bulk}}}}$), and the Curie temperature (Tc) on the heat load cooling was conducted. A reference susceptibility versus temperature graph was constructed. The experimental values of the magnetite (Fe_3_O_4_) nanoparticles χ′ (T) were used as a guide^60^. A Tc of ~ 200 °C was selected to be close to the range of heat load temperatures investigated (Fig. 5a). The results are described in the following subsections.

#### Effect of bulk saturation magnetization on heat load cooling

**Figure 4:**
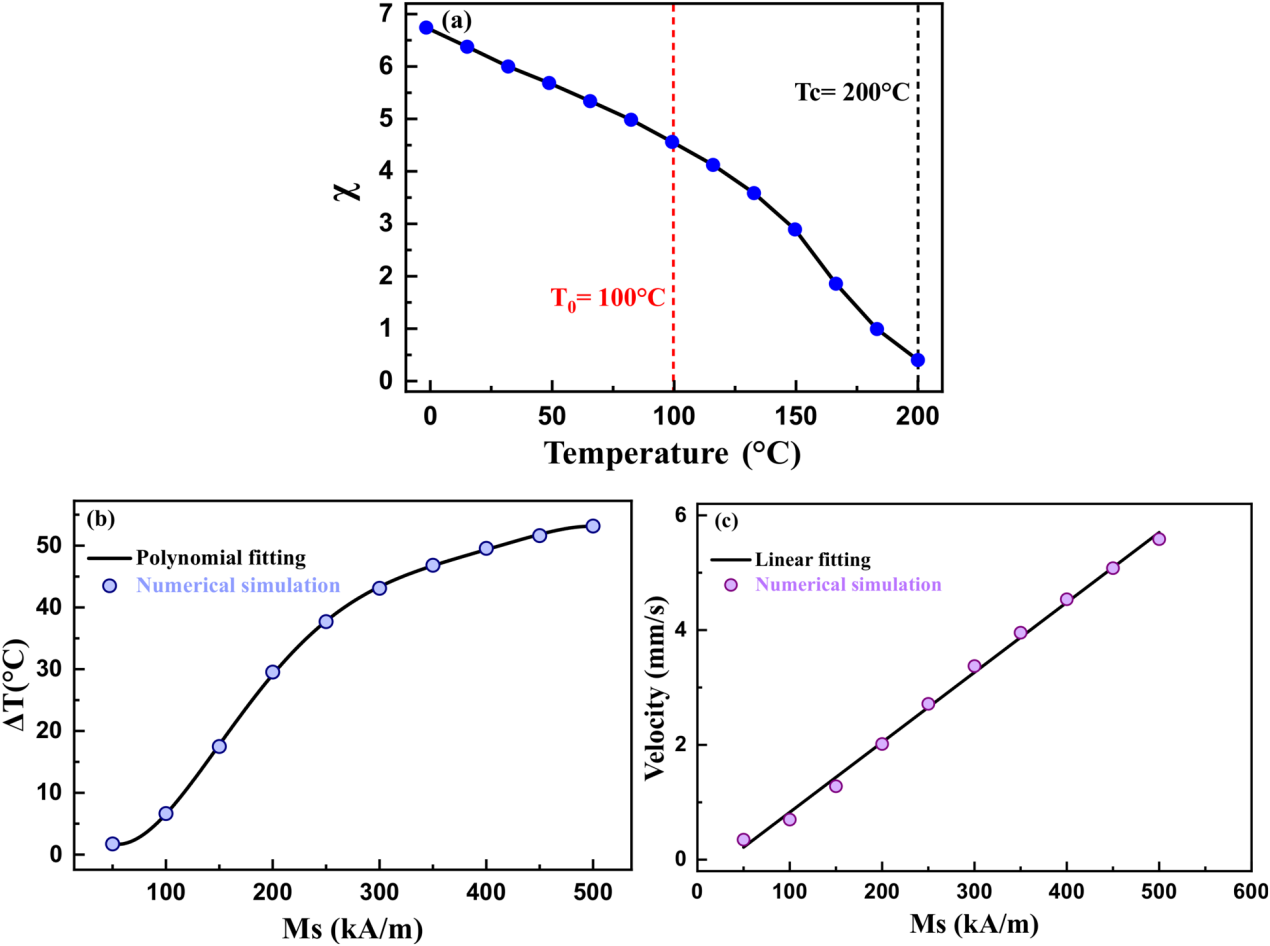
(**a**) Reference initial magnetic susceptibility curve (reference χ_i_ curve), (**b**) the heat load cooling magnitude, and (**c**) the average ferrofluid velocity as a function of the bulk saturation magnetization of the magnetic nanoparticles in the ferrofluid.

For the reference susceptibility curve (Fig. 4a) and an initial heat load temperature of 100 °C, the effect of the ${\text{M}}_{{\text{s}}}^{{{\text{bulk}}}}$ magnetic nanoparticles on heat load cooling performance and average ferrofluid velocity were investigated, as shown in Fig. 4b, c. ΔT increased significantly from 2 °C to 52 °C with an increase of Ms from 50 to 500 kA/m. It followed a polynomial behavior. The average ferrofluid velocity also increased significantly in a linear manner as a function of bulk Ms. The higher value of bulk saturation magnetization results in larger magnetic pressure (Eq. (26)), which gives rise to higher ferrofluid velocity, and hence better cooling.

#### Effect of change in initial susceptibility on heat load cooling

**Figure 5:**
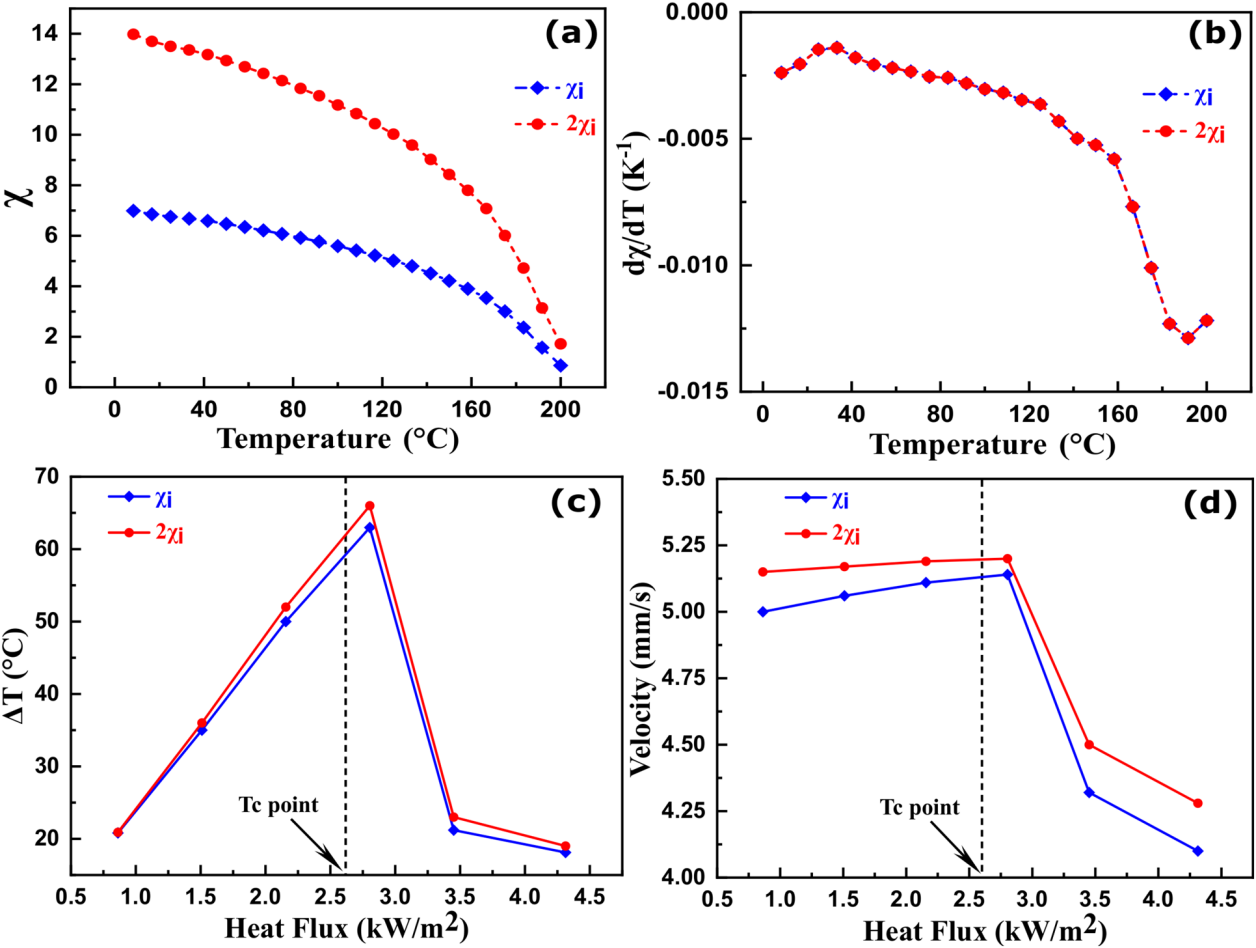
(**a**) Two reference initial magnetic susceptibility curves with different magnitudes, and (**b**) the corresponding pyromagnetic coefficient curves for a Curie temperature of 200 °C. (**c**) The heat load cooling magnitude, and (d) the average ferrofluid velocity as a function of applied heat flux for the virtual magnetic susceptibilities of Fig. 5a, b at a bulk saturation magnetization value of 446 kA/m.

The effect of initial magnetic susceptibility (χ_i_) on the heat load cooling was investigated by assuming two different χ_i_ profiles with the same pyromagnetic coefficient and same Curie temperature of 200 °C (Fig. 5a,b). These susceptibility profiles were provided as inputs to the numerical model. Subsequently, the effects of heat flux and the bulk saturation magnetization on the cooling performance were investigated for a heat load temperature of 200 °C.

Figure 5c, d shows the effect of heat flux on the heat load cooling and the average ferrofluid velocity for these two susceptibility profiles for a bulk saturation magnetization value of 446 kA/m. The heat load temperature was 200 °C. Both the heat load cooling and average ferrofluid velocity increased with larger heat flux values. However, for temperatures higher than the Tc of the ferrofluid, cooling and the velocity reduced sharply. This ΔT reduction is due to the drastic reduction of the thermomagnetic force in the paramagnetic regime of the ferrofluid. The increased cooling for higher heat flux values (below Tc) reveals the self-regulating nature of such magnetic cooling devices.

**Figure 6:**
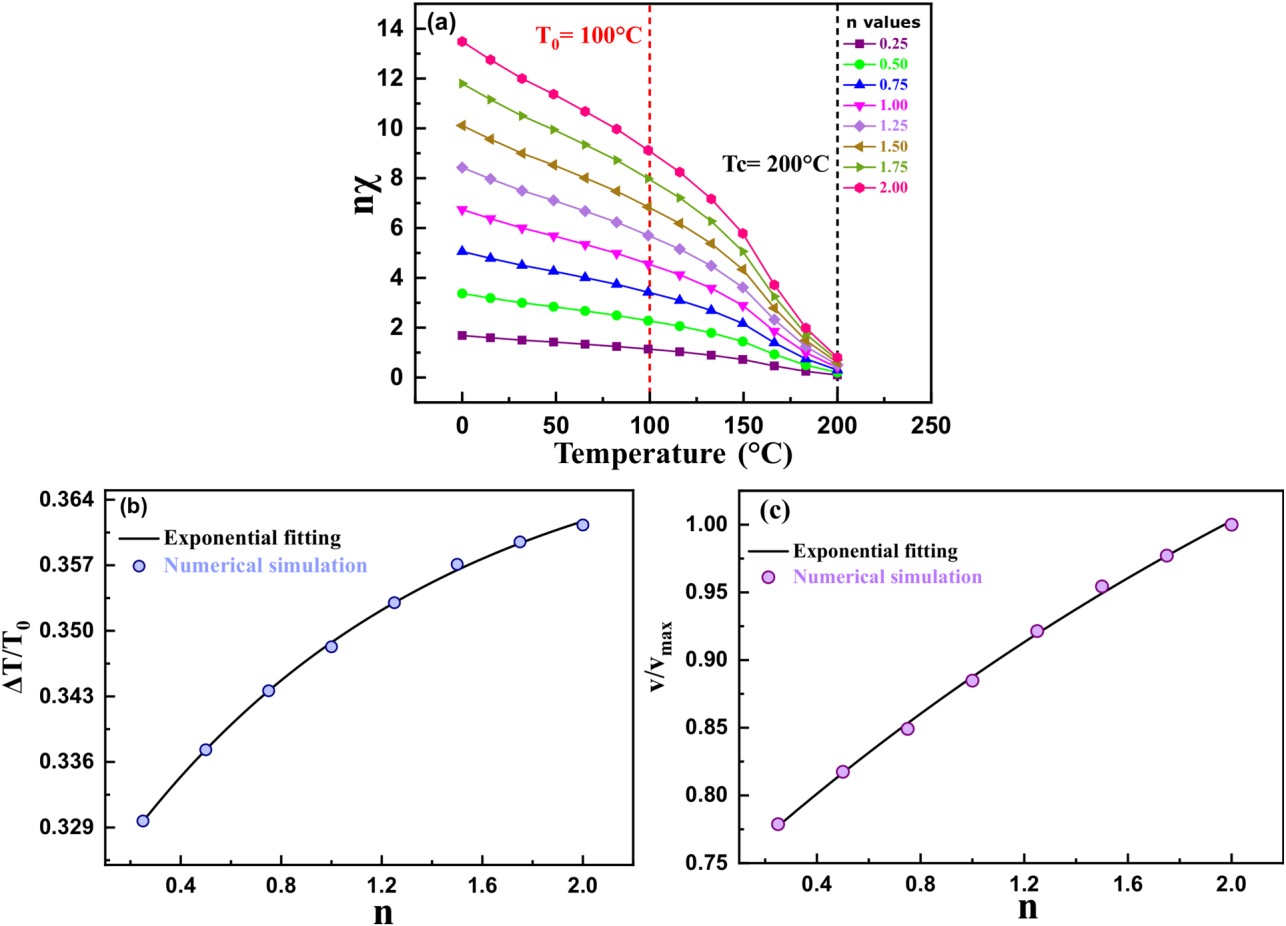
(**a**) Initial magnetic susceptibility profiles with varying magnitudes with respect to the reference susceptibility curve (pink curve). (**b**) Non-dimensional heat load cooling and (**c**) non-dimensional average ferrofluid velocity vs. the ratio of the magnitude of susceptibility to the reference susceptibility curve.

We also investigated the effect of the ratio of initial magnetic susceptibilities to the reference susceptibility profile on the cooling performance (Fig. 6). Various χ_i_ vs. temperature profiles were considered, as shown in Fig. 6a. Both the heat load cooling (Fig. 6b) and the ferrofluid velocity (Fig. 6c) showed a slight increase of 10.6% and 29%, respectively, with a 700% increase in n value (χ″/ χ′, χ′ being the reference χ curve). Hence, initial magnetic susceptibility did not significantly affect cooling.

#### Effect of change in pyromagnetic coefficient on heat load cooling

**Figure 7:**
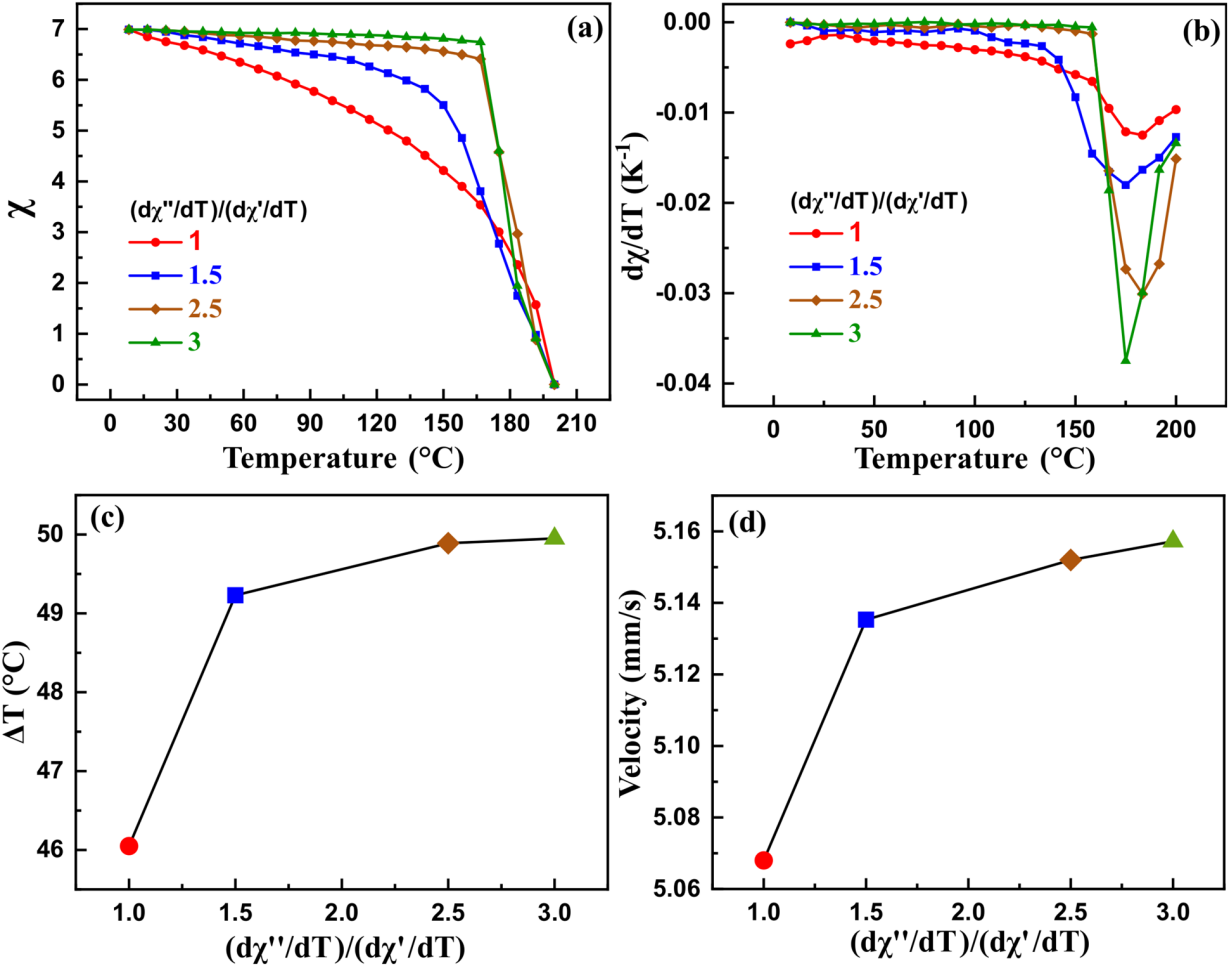
(**a**) Various initial magnetic susceptibility curves with a range of pyromagnetic coefficient ratios, (**b**) the corresponding pyromagnetic coefficient curves at the Curie temperature of 200 °C. (**c**) The heat load cooling magnitude, and (**d**) the average ferrofluid velocity as a function of the ratio of pyromagnetic coefficients curves of Fig. 7. Bulk saturation magnetization is 446 kA/m.

The effect of the pyromagnetic coefficient on the cooling performance was investigated by several virtual susceptibilities vs. temperature curves with increasing pyromagnetic coefficient ratio (Fig. 7a). The corresponding pyromagnetic coefficient vs. temperature curves are shown in Fig. 7b.

The dependence of the heat load cooling magnitude and the average ferrofluid velocity on the pyromagnetic coefficient ratio is plotted in Fig. 7c, d. Both the curves exhibited an increasing trend and showed similar behavior. However, the cooling performance tends to saturate for a higher ratio of the pyromagnetic coefficient. The heat load cooling magnitude increased by only 4 °C for a 200% increase in the pyromagnetic coefficient. Hence, the pyromagnetic coefficient is also not a significant parameter to enhance the cooling.

#### Effect of Curie temperature on heat load cooling

**Figure 8:**
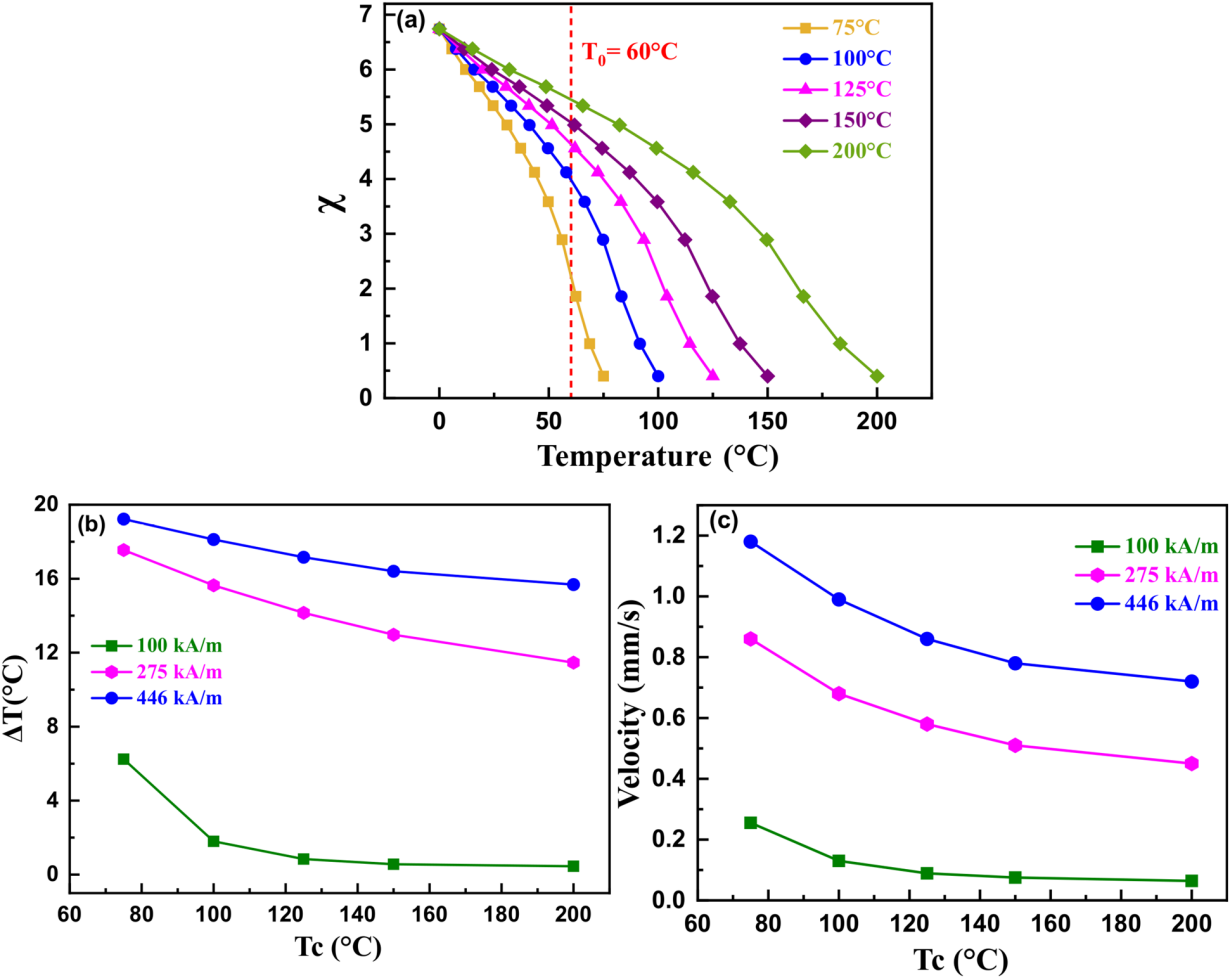
(**a**) Virtual initial magnetic susceptibility curves with varying Curie temperature at same initial susceptibility; (**b**) the heat load cooling magnitude, and (**c**) the average ferrofluid velocity as a function of Curie temperature for a heat load temperature of 60 °C and a bulk saturation magnetization value of 446 kA/m.

To study the effect of Curie temperature (Tc) on magnetic cooling of the heat load, we considered several virtual χ_i_ vs. temperature curves with the same values of M_s_ and initial χ_i_ (Fig. 8a). The effects of Tc on heat load cooling and the ferrofluid velocity profile were studied for a heat load temperature of 60 °C and for a range of bulk saturation magnetization values (Fig. 8b,c). Both of these parameters exhibited an increase in the cooling for values of Tc closer to the heat load temperature. In the present case, a Tc value of 75 °C is closest to the heat load temperature; hence, it exhibited maximum cooling performance (Fig. 8b) as well as the highest velocity (Fig. 8c). These results can be attributed to the high pyromagnetic coefficient (dχ/dt|_T=60 °C_) when the Tc is closer to the heat load temperature. This result is consistent with the effect of the pyromagnetic coefficient (Fig. 7c,d).

From these results, it is clear that ${\text{M}}_{{\text{s}}}^{{{\text{bulk}}}}$ is one of the most significant parameters to improve cooling**.** For constant ${\text{M}}_{{\text{s}}}^{{{\text{bulk}}}}$, the dχ/dt and Tc affect the cooling. On the other hand, the χ_i_ has a negligible effect on the heat load cooling performance. The effects of these parameters can also be understood from the derived expression of magnetic pressure (Eq. (26)).

### Heat load cooling for ferrite and metallic nanoparticle-based ferrofluids

**Figure 9:**
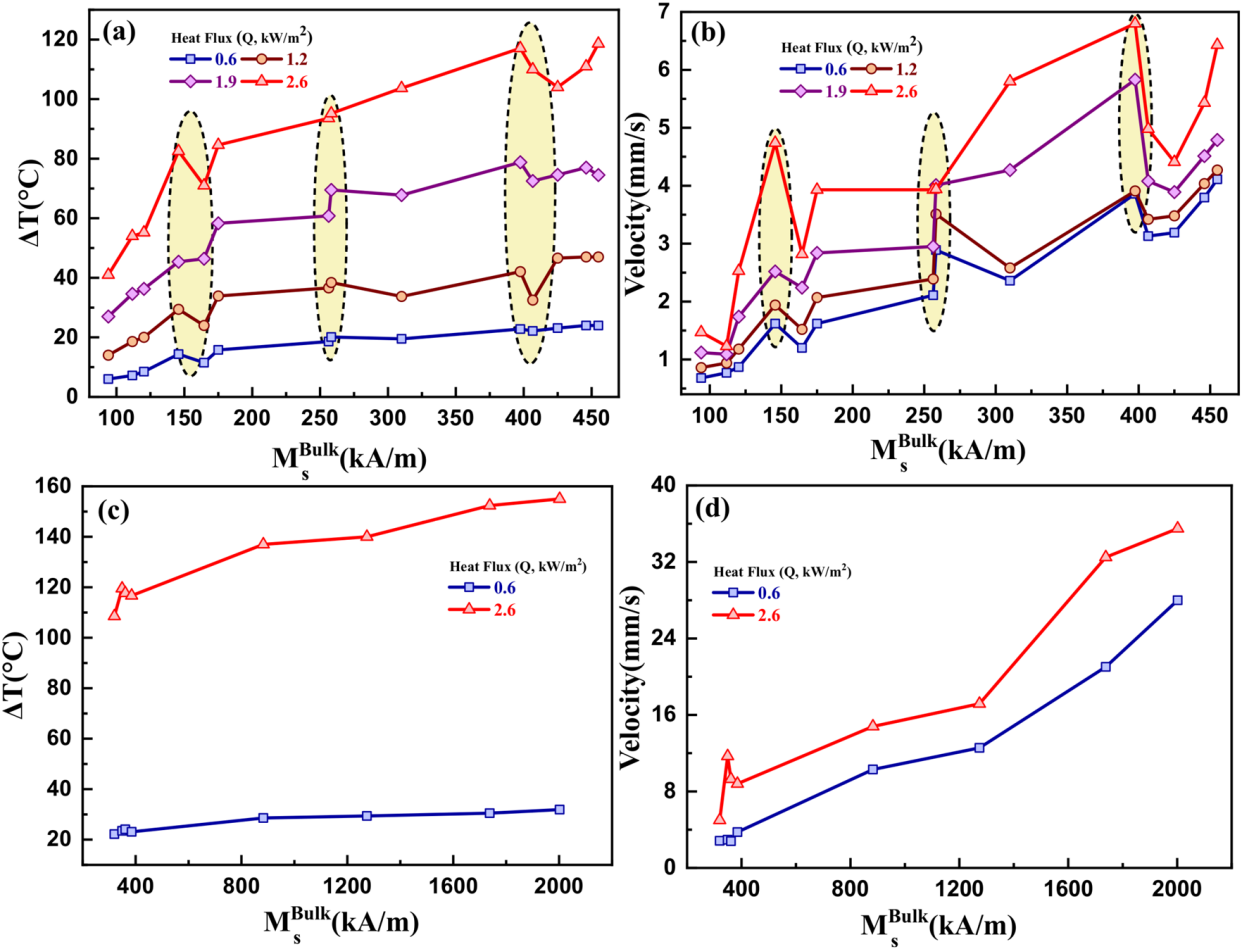
(**a**, **c**) Heat load cooling magnitude and (**b**, **d**) average ferrofluid velocity vs. bulk saturation magnetization of various ferrite and metallic nanoparticle-based ferrofluids.

Numerical simulations were performed using the initial susceptibility vs. temperature curve to rank the ferrite and metallic nanoparticle-based ferrofluids with respect to their thermomagnetic cooling performance. The bulk saturation magnetization of the nanoparticles was provided as input to the model. Figure 9a, b shows the heat load cooling and the average ferrofluid velocity of various ferrite-based ferrofluids as a function of the bulk saturation magnetization of corresponding magnetic nanoparticles. Figure 9c, d is the heat load cooling and the average ferrofluid velocity curves for metallic nanoparticle-based ferrofluids. It shows an increase in heat load cooling and ferrofluid velocity with increasing bulk saturation magnetization for both cases. The cooling and ferrofluid velocity significantly enhanced ferrofluids having higher bulk Ms at high heat flux and heat load temperature.

Certain anomalies were observed for the ΔT and the velocity curves (yellow shaded area) in the case of ferrite-based ferrofluids. These anomalies can be attributed both to the pyromagnetic coefficient and the Curie temperature values. At higher temperatures, the pyromagnetic coefficient is larger. Hence, the anomalies are significant at larger heat load temperatures at higher heat flux values. A decrease (1^st^ and 3^rd^ yellow shaded regions) or increase (2^nd^ and 3^rd^ yellow shaded regions) in cooling and the velocity corresponds to a change in the dχ/dT value. A slight anomaly is observed in the lower bulk Ms region for the metallic ferrofluids case. However, due to the high bulk saturation magnetization of metallic ferrofluids, ΔT and average velocity are significantly larger in comparison to the ferrite ferrofluids. Tables 2 and 3 summarize the cooling performance for various ferrite and metallic ferrofluids, respectively.

**Table 2:** Extent of heat load cooling and average ferrofluid velocity for various ferrite nanoparticle-based ferrofluids.

Materials	Bulk M_s_ (kA/m)	T_0_ = 60 °C, Q = 0.6 kW/m^2^		T_0_ = 100 °C, Q = 1.2 kW/m^2^		T_0_ = 150 °C, Q = 1.9 kW/m^2^		T_0_ = 200 °C, Q = 2.6 kW/m^2^	
		ΔT (°C)	v (mm/s)	ΔT (°C)	v (mm/s)	ΔT (°C)	v (mm/s)	ΔT (°C)	v (mm/s)
Mn_0.3_Zn_0.7_Fe_2_O_4_	94	6	0.68	14	0.86	27	1.12	41	1.47
ZnFe_2_O_4_	111.7	7.2	0.77	18.6	0.94	34.6	1.09	54	1.23
MgFe_2_O_4_	120.2	8.5	0.87	20.02	1.18	36.3	1.74	55.2	2.53
Mn_0.4_Zn_0.6_Fe_2_O_4_	145.7	11.5	1.2	24	1.52	46.4	2.24	71	2.82
Mn_0.5_Zn_0.5_Fe_2_O_4_	164.5	14.35	1.62	29.4	1.94	45.4	2.52	82.5	4.74
NiFe_2_O_4_	175	15.77	1.62	33.9	2.07	58.3	2.84	84.6	3.93
CuFe_2_O_4_	256.3	18.6	2.11	36.6	2.39	60.8	2.95	93.6	3.93
CuNiFe_2_O_4_	258.2	20.1	2.89	38.4	3.51	69.5	4.01	95.2	3.94
Co_0.6_Zn_0.4_Fe_2_O_4_	310	19.5	2.36	33.75	2.58	67.77	4.27	103.7	5.8
γ-Fe_2_O_3_	397.5	22.8	3.86	42.05	3.91	78.8	5.83	117.1	6.8
MnFe_2_O_4_	406.7	22.15	3.13	32.5	3.42	72.5	4.08	110	4.98
Co_0.8_Zn_0.2_Fe_2_O_4_	425	23.1	3.19	46.6	3.48	74.57	3.89	104	4.41
Fe_3_O_4_	446	24	3.796	47	4.036	77	4.512	111	5.43
CoFe_2_O_4_	455	24	4.11	47	4.27	74.5	4.79	118.6	6.43

T_0_ = bare heat load temperature, Q = applied heat flux, ΔT = heat load cooling, v = average ferrofluid velocity.

**Table 3:** Extent of heat load cooling and average ferrofluid velocity for various metallic magnetic nanoparticle-based ferrofluids. Notation: T_0_ = initial heat load temperature, Q = applied heat flux, ΔT = heat load cooling, v = average ferrofluid velocity.

Materials	Bulk M_s_ (kA/m)	T_0_ = 60 °C, Q = 0.6 kW/m^2^		T_0_ = 200 °C, Q = 2.6 kW/m^2^	
		ΔT (°C)	v (mm/s)	ΔT (°C)	v (mm/s)
AlCoFeNiCr	320	22.2	2.84	108.53	5
FeNi_0.7_Cr_0.3_	349	23.5	2.95	119.5	11.69
FeCu	361.2	24.1	2.8	117.67	9.3
FeNi_0.9_Cr_0.1_	385	23.1	3.75	116.7	8.8
AlCoFeNi	882.5	28.6	10.3	137	14.8
Fe_0.3_Ni_0.7_	1274	29.4	12.56	140	17.17
Fe	1738	30.5	21.03	152.4	32.5
FeCo	2002	31.9	28	155	35.5

Hence, at lower heat load temperatures, ferrite ferrofluids having moderate magnetization values exhibit similar cooling performance as their metallic counterparts. On the other hand, at higher heat load temperatures, metallic ferrofluids are more attractive. However, it is usually challenging to synthesize stable metallic magnetic nanoparticle-based ferrofluids due to the oxidation of the metal and the high density of the metal nanoparticles. These issues may lead to particle agglomeration, settling, and a reduction in magnetization of metallic ferrofluids.

### Non-dimensional parameters and figure of merit of ferrofluids

#### Transferred power and exergy loss versus bulk saturation magnetization

**Figure 10:**
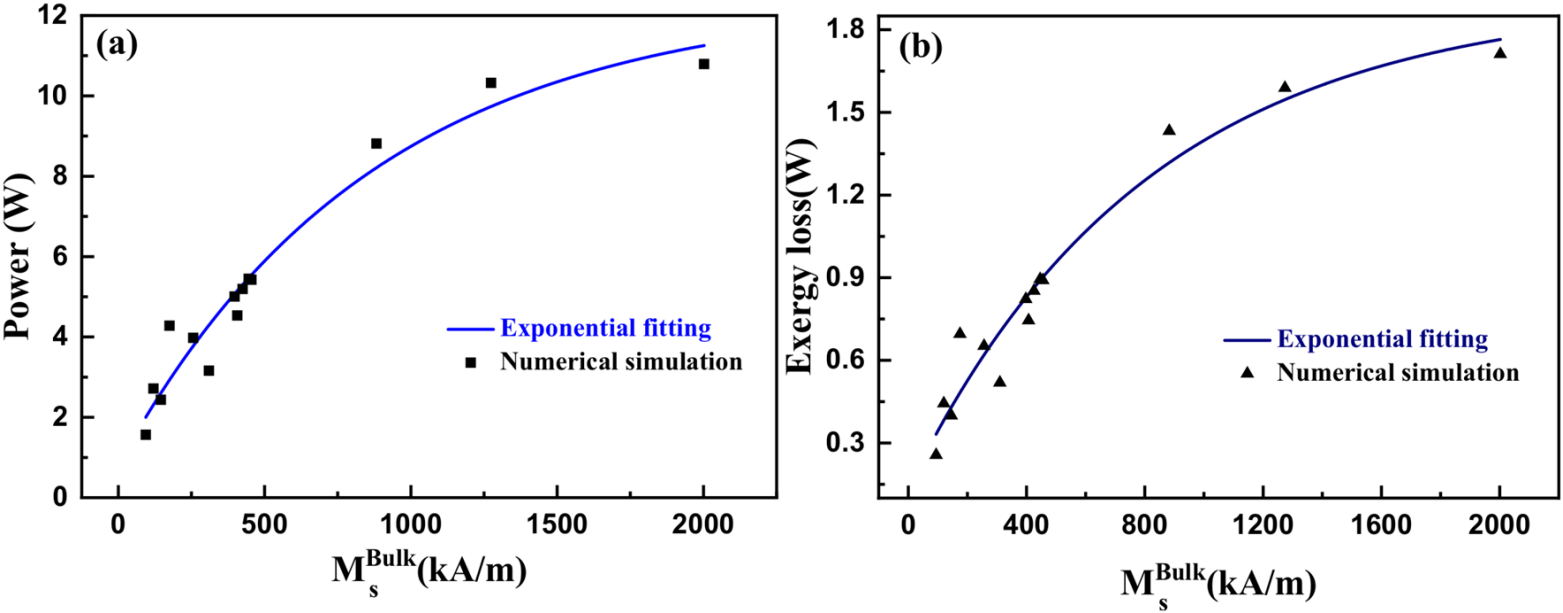
(**a**) Power transferred from heat load to the heat sink and (**b**) exergy loss, as a function of bulk saturation magnetization of various ferrite and metallic nanoparticle-based ferrofluids, for a heat flux value of 1.6 kW/m^2^.

Figure 10a shows a plot of waste heat power vs. bulk saturation magnetization of the magnetic nanoparticles. Waste heat power is the power transported from the heat load to the heat sink by a magnetic cooling device due to the thermomagnetic convection of the ferrofluid. More waste heat power can be removed using highly magnetic ferrofluids. This is due to the higher volume flow rate of ferrofluids having higher magnetization for given magnetic field strength.

Figure 10b shows the exergy loss curve versus the bulk saturation magnetization. The exergy loss also tends to increase with bulk Ms. Exergy loss represents higher randomness. Hence, the entropy maximizes at higher exergy loss. Both the power and the exergy loss are directly proportional to the mass and volume flow rate of the ferrofluid, which is larger for higher bulk Ms due to the greater thermomagnetic volume force experienced by the ferrofluid. The ferrofluid forms vortices near the heat load region due to the combined effect of the thermal gradient and the magnetization gradient^46^. These vortices become prominent for a high magnetization ferrofluid. Hence, the increase in entropy can also be attributed to the formation of strong vortices when the ferrofluid possesses higher magnetization.

#### Non-dimensional parameters versus bulk saturation magnetization

We examined the behavior of several non-dimensional parameters as a function of the bulk saturation magnetization of magnetic nanoparticles to understand the thermomagnetic convection-based heat transfer of a magnetic cooling device.

**Figure 11:**
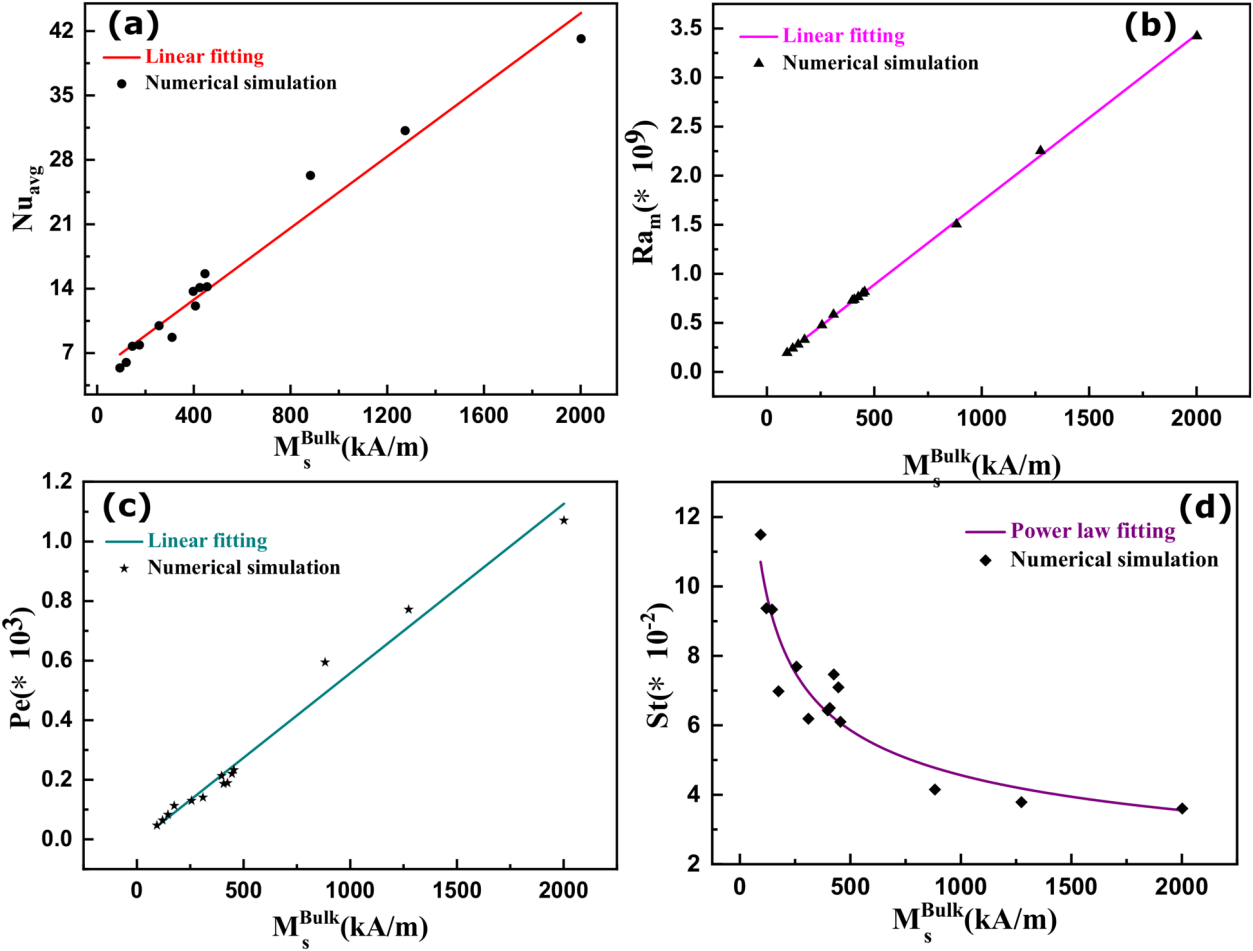
(**a**) The average Nusselt number (Nu_avg_), (**b**) the magnetic Rayleigh number, (**c**) the Peclet number (Pe), and (**c**) the Stanton number (St) as a function of bulk saturation magnetization of various ferrite and metallic nanoparticle-based ferrofluids.

The nature of heat transfer at the heat load region was investigated by calculating the average Nusselt number (Nu_avg_) using Eq. (5), as given in the Supplementary file. Nu_avg_ increases for larger bulk M_s_ (Fig. 11a), implying greater thermomagnetic convection heat transfer from the heat load. This observation is also consistent with the increase in the magnitude of cooling (ΔT) of the heat load for higher bulk M_s_ (Fig. 9). Nu_avg_ is below 10 for bulk M_s_ values below 250 kA/m, suggesting that the ferrofluid flow is laminar. However, for bulk M_s_ values above 250 kA/m, the ferrofluid flow can be considered as a transition between the laminar and turbulent flow regime. The red curve in Fig. 11a suggests a linear increase in the Nu_avg_ with bulk M_s_. Hence, the passive thermomagnetic convection heat transfer increases for ferrofluids having higher saturation magnetization.

The Ra_m_ increases for larger values of bulk Ms (Fig. 11b), which is consistent with Eq. 6 of the Supplementary file. It signifies a stronger dominance of the thermomagnetic force vs. the viscous force for magnetic cooling systems. The Ra_m_ of 10^9^ and above signifies the stronger turbulence effect due to the formation of ferrofluid vortices.

Figure 11c shows the Peclet number (Pe) as a function of bulk M_s_. The linear increase in Pe with increasing bulk M_s_ suggests greater advection heat transfer compared to diffusion-based heat transfer. Thus, the convective motion of the bulk fluid carries a significant amount of waste heat away from the heat load region compared to conduction heat transfer through the nanoparticle chains.

Figure 11d shows the effect of Stanton number (St) as a function of bulk M_s_. The non-linear reduction in St with an increase in bulk M_s_ signifies a reduction in the thermal capacity of the ferrofluid. Conversely, the thermal capacity tends to increase for a higher volume flow rate of the ferrofluid.

**Figure 12:**
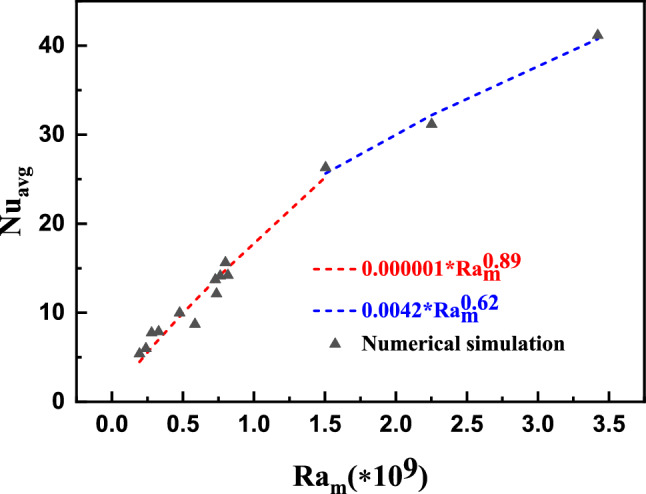
Average Nusselt number (Nu_avg_) versus magnetic Rayleigh number (Ra_m_).

Figure 12 shows the plot of the average Nusselt number against the magnetic Rayleigh number. Nusselt number increases with an increase in Ra_m_ value. As expected, the thermomagnetic force is higher for higher Ra_m_ values, giving rise to a stronger thermomagnetic convection effect, resulting in higher average Nu. However, the average Nu shows two distinct trends. At lower values of Ra_m_, Nu scales as ${\text{Ra}}_{{\text{m}}}^{0.89}$, whereas at higher Ra_m_ values, Nu scales as ${\text{Ra}}_{{\text{m}}}^{0.62}$. This can be attributed to the viscosity of the ferrofluid at the heat load region. When the ferrofluids with higher saturation magnetization (e.g., metallic ferrofluids) are considered, the heat load is cooled significantly compared to lower magnetization ferrofluids. This heat load cooling results in lower ferrofluid temperature at the heat load region for high magnetization ferrofluids compared to low magnetization ferrofluids. Hence, the high magnetization ferrofluids exhibit larger effective viscosity, resulting in lower thermomagnetic convection. This interplay between the magnetization and the viscosity results in two different regimes.

## Conclusions

The cooling performance of the thermomagnetic convection-based magnetic cooling device was numerically assessed with respect to the magnetic properties of various ferrite- and metallic/alloy-nanoparticle-based ferrofluids. For the first time, we ranked the ferrofluids based on their thermomagnetic cooling performance. The effect of bulk saturation magnetization, initial magnetic susceptibility, pyromagnetic coefficient, and the Curie temperature on the heat load cooling was evaluated. Several ferrites- and metallic/alloy-based ferrofluids were simulated to investigate their thermomagnetic cooling performance. Various performance metrics and non-dimensional parameters were evaluated. The conclusions are as follows.

- The bulk saturation magnetization plays the most significant role in cooling the heat load. The initial magnetic susceptibility and pyromagnetic coefficient are less significant.
- Maximum cooling performance is obtained when the Curie temperature is near the heat load temperature.
- γ-Fe_2_O_3_, Fe_3_O_4,_ and CoFe_2_O_4_ ferrofluids displayed better cooling performance than other ferrite-based ferrofluids.
- For metallic/alloy-based ferrofluids, FeCo ferrofluid exhibited the best cooling performance, followed by Fe and FeNi ferrofluids.
- Power transport capability and exergy loss increased for higher bulk saturation magnetization of magnetic nanoparticles.
- The non-dimensional magnetic Rayleigh number, average Nusselt number, and Peclet number confirmed the enhancement in thermomagnetic convection-based passive heat transfer for strongly magnetic ferrofluids.

## Supplementary Information

Supplementary Information.
