# Supplementary material for: Optimal ferrofluids for magnetic cooling devices
Source: Sci Rep. 2021 Dec 17;11:24167. doi: 10.1038/s41598-021-03514-2 (PMC8683428; doi:10.1038/s41598-021-03514-2)
Supplement: Supplementary file 1 — Supplementary Information. [file 41598_2021_3514_MOESM1_ESM.docx]

**Supplementary File**

Optimal Ferrofluids for Magnetic Cooling Devices

M. S. Pattanaik^1,2^, V. B. Varma^1,2^, S. K. Cheekati^1,2^, V. Chaudhary^1^ and R. V. Ramanujan^1,2*^

^1^School of Materials Science and Engineering, Nanyang Technological University, Singapore 639798.

^2^Singapore-HUJ Alliance for Research and Enterprise (SHARE), Nanomaterials for Energy and Energy-Water Nexus (NEW), Campus for Research Excellence and Technological Enterprise (CREATE), Singapore 138602.

# Non-dimensional Numbers

## Nusselt number

Heat transfer using a magnetic cooling device was investigated using the Nusselt number. Nusselt number (Nu) gives the ratio of the convection to conduction heat transfer in a coolant fluid medium. The value of local convective heat transfer coefficient along the flow direction (h_l_) was obtained using the following formula [1],

|  | $h_{l}=\frac{Q_{HL}}{{(T_{w}(l)-T_{f}(l))}_{l}}$ | (1) |
| --- | --- | --- |

where Q_HL_ is the applied heat flux at the heat load. T_w_ and T_f_ are the inner wall temperature of the copper tube and the bulk ferrofluid temperature for a specific position along the flow channel, respectively.

The heat flux can be calculated from the applied power (P), which is the product of the current (I) and voltage (V), divided by the area of the heat load region,

|  | $Q_{HL}=\frac{P_{HL}}{{l_{HL}d}_{t}\pi}=\frac{VI}{{l_{HL}d}_{t}\pi}$ | (2) |
| --- | --- | --- |

The local bulk ferrofluid temperature (T_f_) is given by the equation (3),

|  | $T_{f}\left( l \right)=\frac{{\alpha Q}_{HL}\pi d_{t}}{\kappa q̇}l+T_{in}$ | (3) |
| --- | --- | --- |

The local (${Nu}_{L}$) and the average value (Nu_avg_) of Nusselt number were calculated using equation (4) and equation (5), respectively.

|  | ${Nu}_{L}\left( l \right)=\frac{h_{l}d_{t}}{\kappa}$ | (4) |
| --- | --- | --- |

|  | ${Nu}_{avg}=\sum_{i=1}^{n} \frac{{Nu}_{L}(l)}{n}$ | (5) |
| --- | --- | --- |

where n represents the number of points across the heat load region at which the local Nusselt number was calculated.

## Magnetic Rayleigh number

Magnetic Rayleigh number (Ra_m_) is a dimensionless number, which measures the effects between the magnetic force to the viscous force. Above a critical Ra_m_, magnetic force dominates and leads to the heat transfer by thermomagnetic convection of a ferrofluid. Under the combined effect of magnetization gradient and temperature gradient, a thermomagnetic driving force is established. The thermomagnetic convection can be described by Ra_m_, given by [2],

|  | ${Ra}_{m}= \frac{B_{r}\mathbb{c}_{0}M_{s}^{bulk}l_{LS}}{\eta\alpha}$ | (6) |
| --- | --- | --- |

Here, B_r_ is the residual magnetization of the NdFeB magnet, dt is the characteristic length of the flow channel, η is the dynamic viscosity of the ferrofluid, and α is the thermal diffusivity of the ferrofluid. $l_{\mathrm{LS}}$ represents the characteristic arc length between the heat load to the heat sink, within which the necessary thermal gradient drives the ferrofluid due to the resulting magnetization gradient.

## Peclet number

Convection refers to both advection and diffusion terms. Advection is the transport of heat due to bulk fluid motion, and diffusion is the heat transfer due to the random motion of the molecules [3]. Peclet number (Pe) quantifies the ratio of advection to diffusion during liquid-based heat transfer processes and is given as,

|  | $Pe=\frac{q̇}{\pi d_{t}\alpha}$ | (7) |
| --- | --- | --- |

## Stanton number

Stanton number, also known as the modified Nusselt number, provides a measure of the amount of heat transferred to the fluid from the heat load to the thermal capacity of the fluid. Stanton number can be represented as [3],

|  | $St=\frac{Ah\alpha}{\kappa q̇}=\frac{Nu}{Re*Pr}$ | (8) |
| --- | --- | --- |

Here, Re and Pr represent the Reynolds number and the Prandtl number for the ferrofluid, respectively.

# Numerical Verification and Validation

The developed 2D simulation model was numerically verified by performing a mesh independency test. The non-dimensional heat load cooling (ΔT/ΔT_max_) for a heat flux value of 1.6 kW/m^2^ was plotted as a function of the number of mesh elements (**Figure 1**(a)). The model was also numerically validated by comparing the simulated magnetic field distribution (**Figure 1**(b) and **Figure 1**(c)) results with experimental findings.


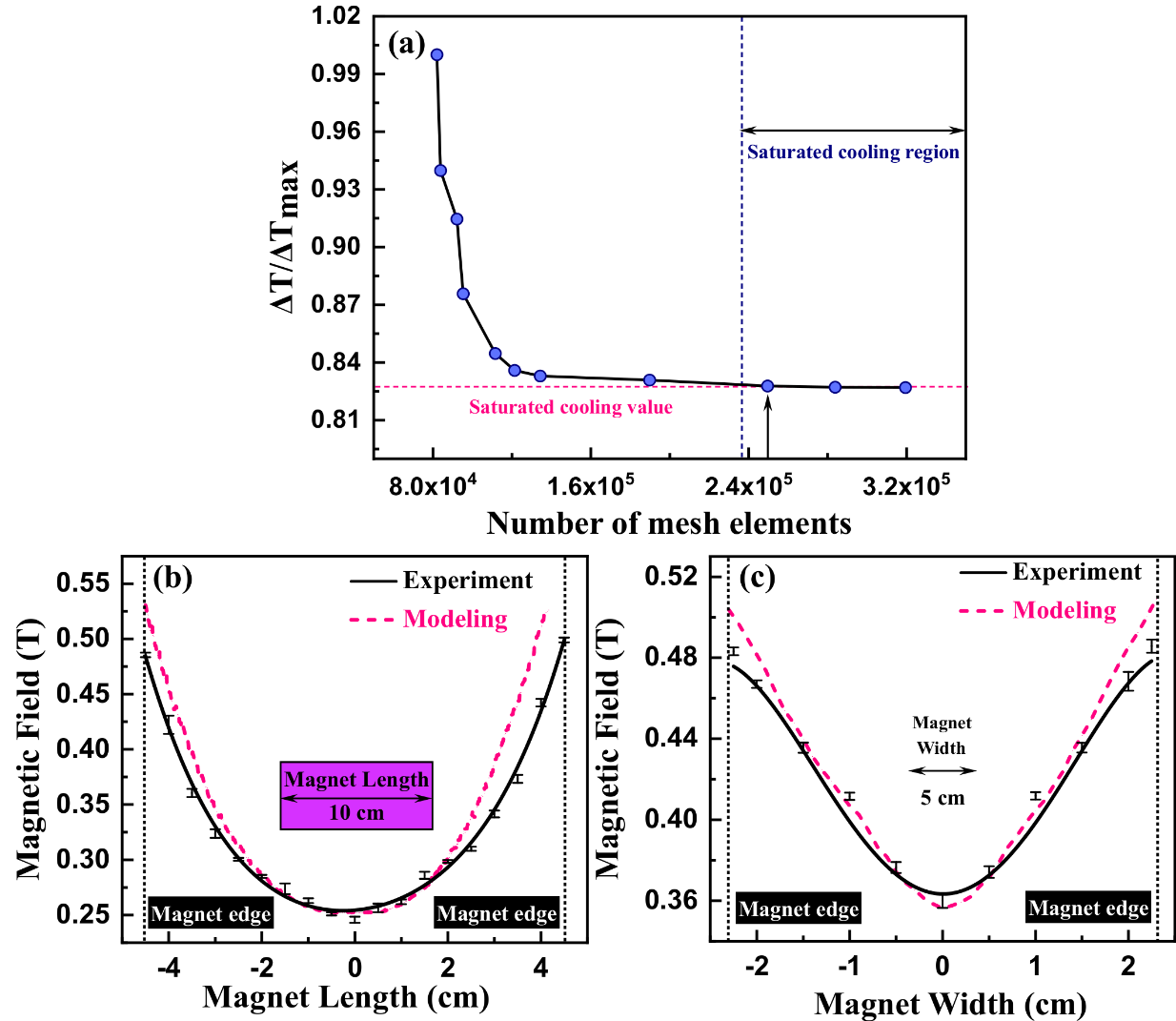


Figure 1. (a) Simulated non-dimensional heat load cooling at an applied heat flux value of 1.6 kW/m^2^ as a function of the number of mesh elements. Experimental and simulated magnetic field distribution along the (b) length and the (c) width of the NdFeB magnet.

# Simulated Surface Temperature and Surface Velocity Vector Plot


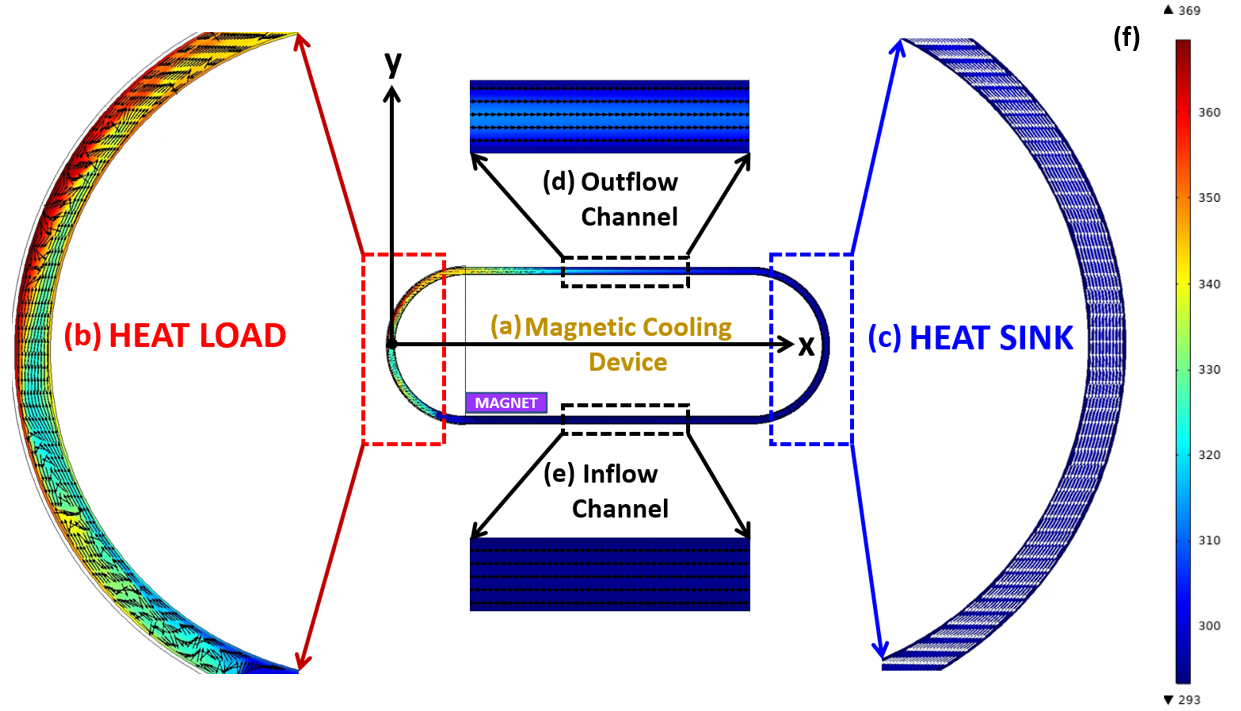


**Figure 2.** (a) Simulated 2D surface temperature and surface velocity vector plots of racetrack shaped magnetic cooling device; zoomed image showing the (b) heat load section, (c) the heat sink section, (d) outflow channel, and (e) inflow channel. (f) the corresponding temperature scale.

**References**

[1] M. A. Khairul, E. Doroodchi, R. Azizian, and B. Moghtaderi, "Thermal performance analysis of tunable magnetite nanofluids for an energy system," *Applied Thermal Engineering,* vol. 126, pp. 822-833, 2017.

[2] M. Ashouri, B. Ebrahimi, M. Shafii, M. Saidi, and M. Saidi, "Correlation for Nusselt number in pure magnetic convection ferrofluid flow in a square cavity by a numerical investigation," *Journal of Magnetism and Magnetic Materials,* vol. 322, no. 22, pp. 3607-3613, 2010.

[3] T. L. Bergman, F. P. Incropera, D. P. DeWitt, and A. S. Lavine, *Fundamentals of heat and mass transfer*. John Wiley & Sons, 2011.
